# Supplementary material for: Transcriptomic Analysis of mRNA Expression Profiles in the Microglia of Mouse Brains Infected with Rabies Viruses of Varying Virulence
Source: Viruses. 2023 May 23;15(6):1223. doi: 10.3390/v15061223 (PMC10303246; doi:10.3390/v15061223)
Supplement: Supplementary file 1 [file viruses-15-01223-s001.zip › Supplemental Table S2 Combined score of Tnf pathway.pdf]

node1	node2	combined_score
Akt3	Mapk13	0.722
Akt3	Mapk14	0.808
Akt3	Atf6b	0.608
Akt3	Map3k8	0.962
Akt3	Casp7	0.565
Akt3	Creb3l1	0.6
Akt3	Creb3l4	0.607
Akt3	Pik3r2	0.947
Akt3	Creb5	0.692
Akt3	Creb3l2	0.608
Akt3	Pik3r1	0.957
Akt3	Mapk11	0.779
Akt3	Mapk12	0.744
Akt3	Casp3	0.785
Akt3	Map3k5	0.991
Akt3	Jun	0.9
Akt3	Atf4	0.619
Akt3	Creb3l3	0.6
Atf4	Atf6b	0.919
Atf4	Fos	0.885
Atf4	Tnf	0.435
Atf4	Rps6ka4	0.803
Atf4	Creb3l1	0.869
Atf4	Creb3l4	0.906
Atf4	Creb5	0.868
Atf4	Creb3l2	0.86
Atf4	Rps6ka5	0.803
Atf4	Cebpb	0.976
Atf4	Casp3	0.606
Atf4	Map3k5	0.532
Atf4	Jun	0.929
Atf4	Creb3l3	0.93
Atf6b	Map3k5	0.428
Atf6b	Creb5	0.807
Atf6b	Rps6ka4	0.846
Atf6b	Rps6ka5	0.851
Atf6b	Creb3l1	0.857
Atf6b	Creb3l4	0.861
Atf6b	Creb3l2	0.862
Atf6b	Creb3l3	0.865
Bcl3	Ccl2	0.412
Bcl3	Mapk14	0.403
Bcl3	Birc3	0.598
Bcl3	Tnfaip3	0.574
Bcl3	Fos	0.433
Bcl3	Traf3	0.506
Bcl3	Map3k8	0.407
Bcl3	Tnf	0.709
Bcl3	Il6	0.535

Bcl3	Il1b	0.632
Bcl3	Tradd	0.437
Bcl3	Cxcl10	0.43
Bcl3	Socs3	0.761
Bcl3	Junb	0.487
Bcl3	Cebpb	0.425
Bcl3	Icam1	0.458
Bcl3	Casp3	0.412
Bcl3	Jun	0.608
Bcl3	Irf1	0.527
Bcl3	Cflar	0.423
Bcl3	Ripk1	0.478
Bcl3	Traf1	0.556
Birc3	Ccl2	0.407
Birc3	Mapk14	0.489
Birc3	Ifnb1	0.401
Birc3	Cxcl2	0.402
Birc3	Map3k8	0.43
Birc3	Ptgs2	0.445
Birc3	Ccl5	0.451
Birc3	Cxcl10	0.452
Birc3	Mmp9	0.5
Birc3	Icam1	0.531
Birc3	Itch	0.577
Birc3	Il6	0.593
Birc3	Jun	0.611
Birc3	Irf1	0.637
Birc3	Il1b	0.662
Birc3	Mlkl	0.777
Birc3	Lta	0.784
Birc3	Map3k5	0.801
Birc3	Nfkbia	0.832
Birc3	Tab3	0.855
Birc3	Fas	0.868
Birc3	Tnfaip3	0.888
Birc3	Tnfrsf1b	0.937
Birc3	Ripk3	0.954
Birc3	Nod2	0.963
Birc3	Cflar	0.966
Birc3	Tnf	0.987
Birc3	Casp7	0.989
Birc3	Fadd	0.989
Birc3	Casp3	0.995
Birc3	Ripk1	0.998
Birc3	Traf1	0.999
Birc3	Tradd	0.999
Birc3	Traf3	0.999
Casp3	Ccl2	0.634
Casp3	Mapk13	0.471
Casp3	Mapk14	0.934

Casp3	Csf1	0.402
Casp3	Mmp9	0.771
Casp3	Map2k3	0.405
Casp3	Nfkbia	0.784
Casp3	Fos	0.602
Casp3	Traf3	0.534
Casp3	Ripk3	0.729
Casp3	Tnf	0.914
Casp3	Fas	0.95
Casp3	Casp7	0.934
Casp3	Il6	0.842
Casp3	Sele	0.421
Casp3	Jag1	0.406
Casp3	Il1b	0.884
Casp3	Vcam1	0.571
Casp3	Fadd	0.986
Casp3	Tradd	0.897
Casp3	Mmp3	0.625
Casp3	Ptgs2	0.717
Casp3	Ccl5	0.479
Casp3	Cxcl10	0.477
Casp3	Mlkl	0.736
Casp3	Ifnb1	0.551
Casp3	Socs3	0.402
Casp3	Cxcl2	0.506
Casp3	Icam1	0.615
Casp3	Mapk11	0.663
Casp3	Mapk12	0.504
Casp3	Map3k5	0.773
Casp3	Traf1	0.776
Casp3	Jun	0.836
Casp3	Cflar	0.837
Casp3	Ripk1	0.963
Casp7	Mapk14	0.607
Casp7	Nfkbia	0.511
Casp7	Ripk3	0.53
Casp7	Tnf	0.7
Casp7	Fas	0.828
Casp7	Il6	0.402
Casp7	Traf1	0.433
Casp7	Jun	0.506
Casp7	Mlkl	0.523
Casp7	Ripk1	0.543
Casp7	Map3k5	0.553
Casp7	Il1b	0.646
Casp7	Tradd	0.741
Casp7	Fadd	0.872
Ccl12	Ccl2	0.883
Ccl12	Mmp9	0.405
Ccl12	Ifnb1	0.411

Ccl12	Junb	0.429
Ccl12	Vcam1	0.469
Ccl12	Icam1	0.499
Ccl12	Csf1	0.53
Ccl12	Fos	0.555
Ccl12	Cxcl3	0.605
Ccl12	Il6	0.687
Ccl12	Tnf	0.69
Ccl12	Cxcl2	0.693
Ccl12	Il1b	0.694
Ccl12	Cxcl10	0.826
Ccl12	Jun	0.912
Ccl2	Mmp14	0.447
Ccl2	Tnfrsf1b	0.51
Ccl2	Junb	0.513
Ccl2	Fas	0.521
Ccl2	Cebpb	0.528
Ccl2	Lif	0.53
Ccl2	Nod2	0.534
Ccl2	Tnfaip3	0.538
Ccl2	Lta	0.587
Ccl2	Irf1	0.602
Ccl2	Socs3	0.659
Ccl2	Mapk14	0.693
Ccl2	Mmp3	0.707
Ccl2	Ifnb1	0.724
Ccl2	Nfkbia	0.726
Ccl2	Sele	0.739
Ccl2	Fos	0.742
Ccl2	Cxcl3	0.748
Ccl2	Il15	0.756
Ccl2	Ptgs2	0.788
Ccl2	Mmp9	0.808
Ccl2	Csf1	0.833
Ccl2	Icam1	0.882
Ccl2	Vcam1	0.894
Ccl2	Cxcl2	0.915
Ccl2	Il1b	0.935
Ccl2	Tnf	0.943
Ccl2	Il6	0.957
Ccl2	Cxcl10	0.961
Ccl2	Jun	0.969
Ccl5	Mapk14	0.552
Ccl5	Csf1	0.73
Ccl5	Mmp9	0.694
Ccl5	Tnfaip3	0.421
Ccl5	Nfkbia	0.64
Ccl5	Tnf	0.908
Ccl5	Lta	0.628
Ccl5	Fas	0.504

Ccl5	Il6	0.91
Ccl5	Sele	0.621
Ccl5	Il1b	0.89
Ccl5	Vcam1	0.766
Ccl5	Tnfrsf1b	0.514
Ccl5	Cxcl3	0.876
Ccl5	Il15	0.827
Ccl5	Mmp3	0.574
Ccl5	Ptgs2	0.608
Ccl5	Lif	0.432
Ccl5	Ifi47	0.466
Ccl5	Nod2	0.476
Ccl5	Socs3	0.498
Ccl5	Traf1	0.57
Ccl5	Irf1	0.698
Ccl5	Ifnb1	0.748
Ccl5	Icam1	0.772
Ccl5	Jun	0.906
Ccl5	Cxcl2	0.944
Ccl5	Cxcl10	0.983
Cebpb	Mapk13	0.869
Cebpb	Mapk14	0.982
Cebpb	Csf1	0.416
Cebpb	Mmp9	0.468
Cebpb	Nfkbia	0.684
Cebpb	Fos	0.868
Cebpb	Tnf	0.731
Cebpb	Il6	0.757
Cebpb	Il1b	0.659
Cebpb	Tradd	0.447
Cebpb	Mmp3	0.403
Cebpb	Ptgs2	0.639
Cebpb	Creb5	0.425
Cebpb	Socs3	0.613
Cebpb	Junb	0.728
Cebpb	Cxcl2	0.424
Cebpb	Irf1	0.567
Cebpb	Mapk12	0.887
Cebpb	Jun	0.951
Cebpb	Mapk11	0.953
Cflar	Mapk14	0.542
Cflar	Mmp9	0.411
Cflar	Tnfaip3	0.507
Cflar	Nfkbia	0.683
Cflar	Traf3	0.51
Cflar	Ripk3	0.852
Cflar	Tnf	0.812
Cflar	Fas	0.992
Cflar	Il6	0.538
Cflar	Il1b	0.725

Cflar	Tnfrsf1b	0.45
Cflar	Fadd	0.999
Cflar	Tradd	0.946
Cflar	Ptgs2	0.416
Cflar	Mlkl	0.71
Cflar	Map3k5	0.653
Cflar	Jun	0.558
Cflar	Itch	0.974
Cflar	Traf1	0.907
Cflar	Ripk1	0.993
Creb3l1	Rps6ka4	0.619
Creb3l1	Rps6ka5	0.608
Creb3l1	Creb5	0.822
Creb3l1	Creb3l2	0.833
Creb3l1	Creb3l4	0.863
Creb3l1	Creb3l3	0.909
Creb3l2	Rps6ka4	0.621
Creb3l2	Creb3l4	0.871
Creb3l2	Creb5	0.836
Creb3l2	Rps6ka5	0.6
Creb3l2	Creb3l3	0.877
Creb3l3	Rps6ka4	0.6
Creb3l3	Creb3l4	0.829
Creb3l3	Creb5	0.816
Creb3l3	Rps6ka5	0.6
Creb3l3	Jun	0.409
Creb3l4	Rps6ka4	0.6
Creb3l4	Jun	0.414
Creb3l4	Rps6ka5	0.6
Creb3l4	Creb5	0.883
Creb5	Mapk13	0.441
Creb5	Mapk14	0.506
Creb5	Fos	0.802
Creb5	Tnf	0.404
Creb5	Rps6ka4	0.607
Creb5	Mapk11	0.464
Creb5	Mapk12	0.469
Creb5	Junb	0.52
Creb5	Rps6ka5	0.607
Creb5	Jun	0.909
Csf1	Mapk14	0.491
Csf1	Mmp3	0.402
Csf1	Lta	0.409
Csf1	Irf1	0.455
Csf1	Lif	0.475
Csf1	Pik3r1	0.485
Csf1	Socs3	0.491
Csf1	Map3k5	0.51
Csf1	Jun	0.53
Csf1	Nfkbia	0.543

Csf1	Fos	0.552
Csf1	Ptgs2	0.553
Csf1	Ifnb1	0.584
Csf1	Icam1	0.585
Csf1	Vcam1	0.587
Csf1	Il15	0.623
Csf1	Mmp9	0.631
Csf1	Cxcl2	0.633
Csf1	Cxcl10	0.725
Csf1	Il1b	0.756
Csf1	Tnf	0.831
Csf1	Il6	0.859
Cxcl10	Mapk14	0.648
Cxcl10	Mmp9	0.635
Cxcl10	Map2k3	0.74
Cxcl10	Tnfaip3	0.606
Cxcl10	Map2k6	0.715
Cxcl10	Nfkbia	0.637
Cxcl10	Traf3	0.4
Cxcl10	Tnf	0.912
Cxcl10	Lta	0.665
Cxcl10	Fas	0.515
Cxcl10	Il6	0.914
Cxcl10	Sele	0.569
Cxcl10	Il1b	0.91
Cxcl10	Vcam1	0.784
Cxcl10	Tnfrsf1b	0.461
Cxcl10	Cxcl3	0.887
Cxcl10	Il15	0.83
Cxcl10	Mmp3	0.528
Cxcl10	Ptgs2	0.625
Cxcl10	Traf1	0.402
Cxcl10	Lif	0.413
Cxcl10	Mlkl	0.423
Cxcl10	Nod2	0.497
Cxcl10	Jun	0.557
Cxcl10	Socs3	0.58
Cxcl10	Ifi47	0.717
Cxcl10	Icam1	0.776
Cxcl10	Ifnb1	0.857
Cxcl10	Irf1	0.893
Cxcl10	Cxcl2	0.955
Cxcl2	Mapk14	0.665
Cxcl2	Mmp9	0.708
Cxcl2	Map2k3	0.413
Cxcl2	Tnfaip3	0.695
Cxcl2	Nfkbia	0.727
Cxcl2	Fos	0.401
Cxcl2	Tnf	0.907
Cxcl2	Il6	0.964

Cxcl2	Sele	0.572
Cxcl2	Il1b	0.971
Cxcl2	Vcam1	0.676
Cxcl2	Cxcl3	0.899
Cxcl2	Il15	0.637
Cxcl2	Mmp3	0.501
Cxcl2	Ptgs2	0.739
Cxcl2	Nod2	0.491
Cxcl2	Ifnb1	0.609
Cxcl2	Socs3	0.589
Cxcl2	Irf1	0.439
Cxcl2	Icam1	0.747
Cxcl2	Jun	0.907
Cxcl3	Mmp9	0.52
Cxcl3	Tnfaip3	0.435
Cxcl3	Nfkbia	0.453
Cxcl3	Tnf	0.622
Cxcl3	Il6	0.834
Cxcl3	Il1b	0.807
Cxcl3	Vcam1	0.412
Cxcl3	Icam1	0.403
Cxcl3	Il15	0.465
Cxcl3	Ptgs2	0.576
Cxcl3	Jun	0.856
Fadd	Mapk14	0.463
Fadd	Tnfaip3	0.966
Fadd	Nfkbia	0.56
Fadd	Traf3	0.797
Fadd	Ripk3	0.999
Fadd	Tnf	0.996
Fadd	Lta	0.524
Fadd	Fas	0.999
Fadd	Il6	0.465
Fadd	Il1b	0.621
Fadd	Tnfrsf1b	0.415
Fadd	Ifnb1	0.524
Fadd	Jun	0.551
Fadd	Map3k5	0.824
Fadd	Traf1	0.858
Fadd	Mlkl	0.912
Fadd	Tradd	0.999
Fadd	Ripk1	0.999
Fas	Mapk14	0.432
Fas	Map2k3	0.67
Fas	Map2k6	0.693
Fas	Nfkbia	0.448
Fas	Traf3	0.403
Fas	Ripk3	0.685
Fas	Tnf	0.836
Fas	Vcam1	0.422

Fas	Ifnb1	0.474
Fas	Traf1	0.482
Fas	Tnfrsf1b	0.486
Fas	Jun	0.499
Fas	Pik3r2	0.556
Fas	Il15	0.603
Fas	Icam1	0.629
Fas	Mlkl	0.631
Fas	Il1b	0.667
Fas	Il6	0.722
Fas	Pik3r1	0.738
Fas	Map3k5	0.862
Fas	Tradd	0.958
Fas	Ripk1	0.969
Fos	Mapk13	0.924
Fos	Mapk14	0.982
Fos	Mmp9	0.641
Fos	Map2k3	0.435
Fos	Nfkbia	0.685
Fos	Map3k5	0.406
Fos	Traf1	0.414
Fos	Icam1	0.431
Fos	Mmp3	0.441
Fos	Traf3	0.478
Fos	Jag1	0.52
Fos	Pik3r1	0.583
Fos	Irf1	0.61
Fos	Socs3	0.627
Fos	Ptgs2	0.648
Fos	Il1b	0.678
Fos	Ifnb1	0.794
Fos	Il6	0.89
Fos	Tnf	0.891
Fos	Mapk12	0.929
Fos	Mapk11	0.95
Fos	Jun	0.999
Fos	Junb	0.999
Gm5431	Ifi47	0.812
Icam1	Mapk14	0.68
Icam1	Mmp9	0.744
Icam1	Tnfaip3	0.521
Icam1	Nfkbia	0.771
Icam1	Tnf	0.91
Icam1	Lta	0.56
Icam1	Il6	0.863
Icam1	Sele	0.901
Icam1	Il1b	0.889
Icam1	Vcam1	0.946
Icam1	Tnfrsf1b	0.526
Icam1	Il15	0.61

Icam1	Mmp3	0.572
Icam1	Ptgs2	0.711
Icam1	Nod2	0.422
Icam1	Ifnb1	0.585
Icam1	Socs3	0.609
Icam1	Traf1	0.553
Icam1	Irf1	0.606
Icam1	Jun	0.661
Ifi47	Irf1	0.783
Ifnb1	Mapk14	0.557
Ifnb1	Mmp9	0.467
Ifnb1	Tnfaip3	0.401
Ifnb1	Nfkbia	0.688
Ifnb1	Traf3	0.786
Ifnb1	Ripk3	0.658
Ifnb1	Tnf	0.856
Ifnb1	Lta	0.463
Ifnb1	Il6	0.87
Ifnb1	Il1b	0.829
Ifnb1	Vcam1	0.465
Ifnb1	Il15	0.665
Ifnb1	Tradd	0.431
Ifnb1	Ptgs2	0.514
Ifnb1	Nod2	0.687
Ifnb1	Mlkl	0.463
Ifnb1	Junb	0.485
Ifnb1	Socs3	0.813
Ifnb1	Jun	0.876
Ifnb1	Irf1	0.902
Ifnb1	Ripk1	0.926
Il15	Mmp9	0.4
Il15	Tnf	0.855
Il15	Lta	0.697
Il15	Il6	0.844
Il15	Il1b	0.805
Il15	Vcam1	0.68
Il15	Il18r1	0.4
Il15	Ptgs2	0.405
Il15	Socs3	0.475
Il15	Lif	0.477
Il15	Irf1	0.57
Il18r1	Tnf	0.475
Il18r1	Il6	0.463
Il18r1	Il1b	0.687
Il18r1	Junb	0.481
Il18r1	Jun	0.696
Il1b	Mapk13	0.474
Il1b	Mapk14	0.933
Il1b	Mmp9	0.874
Il1b	Map2k3	0.475

Il1b	Tnfaip3	0.842
Il1b	Nfkbia	0.902
Il1b	Traf3	0.781
Il1b	Ripk3	0.768
Il1b	Map3k8	0.551
Il1b	Tnf	0.973
Il1b	Lta	0.765
Il1b	Il6	0.981
Il1b	Sele	0.732
Il1b	Tradd	0.475
Il1b	Map3k5	0.505
Il1b	Mmp14	0.516
Il1b	Mapk12	0.53
Il1b	Pik3r2	0.552
Il1b	Lif	0.568
Il1b	Mlkl	0.61
Il1b	Tnfrsf1b	0.708
Il1b	Irf1	0.724
Il1b	Pik3r1	0.73
Il1b	Traf1	0.739
Il1b	Mapk11	0.75
Il1b	Ripk1	0.759
Il1b	Nod2	0.774
Il1b	Mmp3	0.788
Il1b	Socs3	0.798
Il1b	Vcam1	0.841
Il1b	Ptgs2	0.902
Il1b	Jun	0.962
Il6	Mapk14	0.841
Il6	Mmp9	0.861
Il6	Map2k3	0.746
Il6	Tnfaip3	0.656
Il6	Map2k6	0.768
Il6	Nfkbia	0.873
Il6	Traf3	0.552
Il6	Ripk3	0.501
Il6	Map3k8	0.516
Il6	Tnf	0.947
Il6	Lta	0.731
Il6	Tradd	0.463
Il6	Map3k5	0.465
Il6	Mmp14	0.486
Il6	Traf1	0.501
Il6	Mlkl	0.511
Il6	Ripk1	0.546
Il6	Jag1	0.571
Il6	Tnfrsf1b	0.677
Il6	Junb	0.684
Il6	Irf1	0.689
Il6	Nod2	0.725

Il6	Lif	0.764
Il6	Mmp3	0.772
Il6	Sele	0.775
Il6	Vcam1	0.869
Il6	Ptgs2	0.925
Il6	Socs3	0.982
Il6	Jun	0.988
Irf1	Tnfaip3	0.592
Irf1	Nfkbia	0.621
Irf1	Traf3	0.473
Irf1	Tnf	0.751
Irf1	Vcam1	0.406
Irf1	Ptgs2	0.413
Irf1	Socs3	0.627
Irf1	Junb	0.668
Irf1	Jun	0.918
Irf1	Traf1	0.411
Itch	Jag1	0.568
Itch	Nod2	0.926
Itch	Junb	0.567
Itch	Jun	0.847
Itch	Ripk1	0.775
Jag1	Mmp9	0.47
Jag1	Tnf	0.526
Jag1	Vcam1	0.423
Jag1	Jun	0.652
Jun	Mapk13	0.944
Jun	Mapk14	0.995
Jun	Mmp9	0.782
Jun	Map2k3	0.721
Jun	Tnfaip3	0.428
Jun	Map2k6	0.592
Jun	Nfkbia	0.937
Jun	Traf3	0.545
Jun	Map3k8	0.469
Jun	Tnf	0.989
Jun	Lta	0.509
Jun	Sele	0.499
Jun	Vcam1	0.589
Jun	Tradd	0.639
Jun	Mmp3	0.679
Jun	Ptgs2	0.743
Jun	Nod2	0.444
Jun	Socs3	0.577
Jun	Junb	0.969
Jun	Mapk11	0.975
Jun	Mapk12	0.976
Jun	Mmp14	0.499
Jun	Map3k5	0.803
Jun	Traf1	0.502

Jun	Ripk1	0.54
Junb	Mapk13	0.78
Junb	Mapk14	0.842
Junb	Nfkbia	0.523
Junb	Tnf	0.656
Junb	Socs3	0.677
Junb	Mapk12	0.783
Junb	Mapk11	0.796
Lif	Tnf	0.592
Lif	Socs3	0.561
Lta	Tnfaip3	0.452
Lta	Nfkbia	0.432
Lta	Traf3	0.801
Lta	Tnf	0.841
Lta	Tab3	0.457
Lta	Traf1	0.504
Lta	Tradd	0.659
Lta	Vcam1	0.72
Lta	Tnfrsf1b	0.967
Map2k3	Mapk13	0.971
Map2k3	Mapk14	0.996
Map2k3	Ripk3	0.41
Map2k3	Mlkl	0.443
Map2k3	Nfkbia	0.48
Map2k3	Traf1	0.589
Map2k3	Traf3	0.768
Map2k3	Tnf	0.852
Map2k3	Nod2	0.908
Map2k3	Ripk1	0.945
Map2k3	Tradd	0.956
Map2k3	Tab3	0.958
Map2k3	Mapk12	0.974
Map2k3	Map2k6	0.984
Map2k3	Map3k5	0.988
Map2k3	Mapk11	0.991
Map2k6	Mapk13	0.983
Map2k6	Mapk14	0.996
Map2k6	Mlkl	0.409
Map2k6	Traf3	0.492
Map2k6	Traf1	0.565
Map2k6	Tnf	0.796
Map2k6	Nod2	0.91
Map2k6	Tradd	0.916
Map2k6	Ripk1	0.934
Map2k6	Tab3	0.959
Map2k6	Mapk12	0.986
Map2k6	Map3k5	0.989
Map2k6	Mapk11	0.991
Map3k5	Mapk13	0.832
Map3k5	Mapk14	0.875

Map3k5	Nfkbia	0.44
Map3k5	Traf3	0.516
Map3k5	Ripk3	0.486
Map3k5	Tnf	0.893
Map3k5	Tradd	0.884
Map3k5	Mlkl	0.558
Map3k5	Mapk11	0.859
Map3k5	Mapk12	0.84
Map3k5	Ripk1	0.78
Map3k5	Traf1	0.879
Map3k8	Tnfaip3	0.582
Map3k8	Nfkbia	0.588
Map3k8	Tab3	0.401
Map3k8	Traf1	0.492
Map3k8	Tnf	0.593
Map3k8	Ripk1	0.621
Mapk11	Mapk13	0.904
Mapk11	Mapk14	0.832
Mapk11	Nfkbia	0.673
Mapk11	Tnf	0.792
Mapk11	Rps6ka4	0.971
Mapk11	Tab3	0.725
Mapk11	Rps6ka5	0.981
Mapk11	Traf1	0.453
Mapk11	Ripk1	0.609
Mapk11	Mapk12	0.909
Mapk12	Mapk13	0.919
Mapk12	Mapk14	0.922
Mapk12	Nfkbia	0.541
Mapk12	Tnf	0.584
Mapk12	Rps6ka4	0.871
Mapk12	Tab3	0.756
Mapk12	Rps6ka5	0.879
Mapk12	Ripk1	0.438
Mapk13	Ripk1	0.443
Mapk13	Tnf	0.462
Mapk13	Nfkbia	0.524
Mapk13	Tab3	0.751
Mapk13	Rps6ka4	0.871
Mapk13	Rps6ka5	0.886
Mapk13	Mapk14	0.903
Mapk14	Nod2	0.428
Mapk14	Mlkl	0.449
Mapk14	Traf3	0.457
Mapk14	Tradd	0.463
Mapk14	Mmp3	0.534
Mapk14	Sele	0.537
Mapk14	Traf1	0.59
Mapk14	Socs3	0.604
Mapk14	Vcam1	0.632

Mapk14	Mmp9	0.703
Mapk14	Ptgs2	0.753
Mapk14	Ripk1	0.758
Mapk14	Tab3	0.809
Mapk14	Tnf	0.94
Mapk14	Nfkbia	0.941
Mapk14	Rps6ka5	0.985
Mapk14	Rps6ka4	0.985
Mlkl	Nfkbia	0.448
Mlkl	Ripk3	0.993
Mlkl	Tnf	0.776
Mlkl	Tnfrsf1b	0.448
Mlkl	Tradd	0.829
Mlkl	Tab3	0.438
Mlkl	Ripk1	0.815
Mmp14	Tnf	0.576
Mmp14	Ptgs2	0.41
Mmp14	Pik3r1	0.518
Mmp3	Mmp9	0.919
Mmp3	Nfkbia	0.548
Mmp3	Tnf	0.773
Mmp3	Sele	0.451
Mmp3	Vcam1	0.554
Mmp3	Ptgs2	0.687
Mmp9	Socs3	0.533
Mmp9	Sele	0.622
Mmp9	Nfkbia	0.716
Mmp9	Vcam1	0.733
Mmp9	Ptgs2	0.752
Mmp9	Tnf	0.876
Nfkbia	Tnfaip3	0.909
Nfkbia	Rps6ka5	0.401
Nfkbia	Tnfrsf1b	0.439
Nfkbia	Ripk3	0.53
Nfkbia	Sele	0.563
Nfkbia	Nod2	0.575
Nfkbia	Pik3r2	0.617
Nfkbia	Ripk1	0.683
Nfkbia	Vcam1	0.684
Nfkbia	Socs3	0.729
Nfkbia	Traf3	0.737
Nfkbia	Tradd	0.766
Nfkbia	Ptgs2	0.789
Nfkbia	Tab3	0.831
Nfkbia	Pik3r1	0.835
Nfkbia	Traf1	0.883
Nfkbia	Tnf	0.971
Nod2	Tnfaip3	0.949
Nod2	Traf3	0.474
Nod2	Ripk3	0.469

Nod2	Tnf	0.745
Nod2	Tab3	0.733
Nod2	Ripk1	0.568
Nod2	Socs3	0.642
Pik3r1	Pik3r2	0.952
Pik3r1	Socs3	0.676
Pik3r2	Socs3	0.6
Ptgs2	Tnfaip3	0.486
Ptgs2	Tnf	0.869
Ptgs2	Sele	0.569
Ptgs2	Vcam1	0.683
Ptgs2	Socs3	0.526
Ripk1	Tnfaip3	0.995
Ripk1	Traf3	0.891
Ripk1	Ripk3	0.997
Ripk1	Tnf	0.998
Ripk1	Tnfrsf1b	0.787
Ripk1	Tradd	0.999
Ripk1	Tab3	0.936
Ripk1	Traf1	0.981
Ripk3	Tnfaip3	0.886
Ripk3	Traf3	0.422
Ripk3	Traf1	0.445
Ripk3	Tab3	0.454
Ripk3	Tnfrsf1b	0.486
Ripk3	Tnf	0.805
Ripk3	Tradd	0.989
Rps6ka4	Rps6ka5	0.933
Sele	Tnf	0.843
Sele	Vcam1	0.916
Socs3	Tnfaip3	0.489
Socs3	Tnf	0.747
Tab3	Tnfaip3	0.93
Tab3	Traf3	0.947
Tab3	Tnf	0.869
Tab3	Tradd	0.901
Tab3	Traf1	0.535
Tnf	Tnfaip3	0.995
Tnf	Traf3	0.932
Tnf	Vcam1	0.953
Tnf	Traf1	0.982
Tnf	Tradd	0.999
Tnf	Tnfrsf1b	0.999
Tnfaip3	Tnfrsf1b	0.422
Tnfaip3	Traf3	0.658
Tnfaip3	Tradd	0.913
Tnfaip3	Traf1	0.99
Tnfrsf1b	Traf3	0.932
Tnfrsf1b	Vcam1	0.414
Tnfrsf1b	Tradd	0.89

Tnfrsf1b	Traf1	0.983
Tradd	Traf3	0.993
Tradd	Traf1	0.996
Traf1	Traf3	0.951
