# Supplementary material for: Transcriptomic Analysis of mRNA Expression Profiles in the Microglia of Mouse Brains Infected with Rabies Viruses of Varying Virulence
Source: Viruses. 2023 May 23;15(6):1223. doi: 10.3390/v15061223 (PMC10303246; doi:10.3390/v15061223)
Supplement: Supplementary file 1 [file viruses-15-01223-s001.zip › Supplemental Table S3 Combined score of Tlr pathway.pdf]

Tlr pathway

node1	node2	combined_score
Akt3	Cd40	0.555
Akt3	Map3k8	0.962
Akt3	Creb3l2	0.608
Akt3	Ikbke	0.553
Akt3	Pik3r1	0.957
Akt3	Mapk11	0.779
Akt3	Mapk12	0.744
Akt3	Jun	0.9
Bcl3	Cd40	0.673
Bcl3	Fos	0.433
Bcl3	Traf3	0.506
Bcl3	Map3k8	0.407
Bcl3	Tnf	0.709
Bcl3	Il1b	0.632
Bcl3	Tlr2	0.509
Bcl3	Myd88	0.477
Bcl3	Cxcl10	0.43
Bcl3	Ikbke	0.644
Bcl3	Stat1	0.517
Bcl3	Jun	0.608
Ccl12	Mmp9	0.405
Ccl12	Ifnb1	0.411
Ccl12	Cd40	0.434
Ccl12	Tlr2	0.462
Ccl12	Ccl4	0.514
Ccl12	Csf1	0.53
Ccl12	Fos	0.555
Ccl12	Tnf	0.69
Ccl12	Il1b	0.694
Ccl12	Cxcl9	0.753
Ccl12	Cxcl10	0.826
Ccl12	Jun	0.912
Ccl3	Ifna2	0.405
Ccl3	Tlr5	0.475
Ccl3	Irf7	0.491
Ccl3	Jun	0.511
Ccl3	Tlr8	0.534
Ccl3	Spp1	0.538
Ccl3	Nfkbia	0.551
Ccl3	Stat1	0.561
Ccl3	Tlr3	0.62
Ccl3	Tlr9	0.621
Ccl3	Mmp9	0.628
Ccl3	Myd88	0.655
Ccl3	Cd40	0.688
Ccl3	Ifnb1	0.69
Ccl3	Cd14	0.711
Ccl3	Csf1	0.724

Ccl3	Il12b	0.757
Ccl3	Tlr2	0.81
Ccl3	Cxcl9	0.854
Ccl3	Ccl4	0.918
Ccl3	Cxcl10	0.931
Ccl3	Tnf	0.934
Ccl3	Il1b	0.947
Ccl4	Csf1	0.704
Ccl4	Cd40	0.67
Ccl4	Mmp9	0.565
Ccl4	Ifna2	0.4
Ccl4	Spp1	0.429
Ccl4	Tlr5	0.43
Ccl4	Nfkbia	0.523
Ccl4	Tlr8	0.524
Ccl4	Irf7	0.559
Ccl4	Tlr9	0.569
Ccl4	Myd88	0.581
Ccl4	Stat1	0.582
Ccl4	Tlr3	0.593
Ccl4	Ifnb1	0.653
Ccl4	Cd14	0.678
Ccl4	Fos	0.733
Ccl4	Il12b	0.746
Ccl4	Tlr2	0.768
Ccl4	Jun	0.781
Ccl4	Ccl5	0.782
Ccl4	Tnf	0.948
Ccl4	Il1b	0.954
Ccl4	Cxcl9	0.955
Ccl4	Cxcl10	0.988
Ccl5	Csf1	0.73
Ccl5	Cd40	0.745
Ccl5	Mmp9	0.694
Ccl5	Nfkbia	0.64
Ccl5	Tnf	0.908
Ccl5	Irf7	0.772
Ccl5	Il1b	0.89
Ccl5	Tlr2	0.774
Ccl5	Tlr3	0.728
Ccl5	Myd88	0.709
Ccl5	Ticam2	0.4
Ccl5	Ifi47	0.466
Ccl5	Spp1	0.499
Ccl5	Ikbke	0.532
Ccl5	Tlr5	0.547
Ccl5	Tlr8	0.547
Ccl5	Cd14	0.612
Ccl5	Tlr9	0.647
Ccl5	Ifnb1	0.748

Ccl5	Stat1	0.768
Ccl5	Il12b	0.772
Ccl5	Jun	0.906
Ccl5	Cxcl9	0.965
Ccl5	Cxcl10	0.983
Cd14	Csf1	0.662
Cd14	Cd40	0.63
Cd14	Mmp9	0.493
Cd14	Nfkbia	0.509
Cd14	Traf3	0.763
Cd14	Tnf	0.818
Cd14	Irf7	0.732
Cd14	Il1b	0.827
Cd14	Tlr2	0.992
Cd14	Tlr3	0.748
Cd14	Myd88	0.947
Cd14	Cxcl10	0.619
Cd14	Ikbke	0.747
Cd14	Spp1	0.424
Cd14	Cxcl9	0.432
Cd14	Stat1	0.448
Cd14	Jun	0.468
Cd14	Ifnb1	0.561
Cd14	Il12b	0.574
Cd14	Ticam2	0.679
Cd14	Tlr5	0.701
Cd14	Tlr8	0.711
Cd14	Tlr9	0.718
Cd40	Csf1	0.598
Cd40	Mmp9	0.484
Cd40	Map3k8	0.533
Cd40	Ikbke	0.538
Cd40	Il12a	0.552
Cd40	Tlr5	0.56
Cd40	Tlr8	0.562
Cd40	Irf7	0.565
Cd40	Cxcl9	0.639
Cd40	Jun	0.657
Cd40	Ifnb1	0.677
Cd40	Mapk11	0.701
Cd40	Cxcl10	0.735
Cd40	Stat1	0.769
Cd40	Il12b	0.769
Cd40	Tlr2	0.779
Cd40	Myd88	0.794
Cd40	Tlr3	0.794
Cd40	Il1b	0.848
Cd40	Pik3r1	0.854
Cd40	Tlr9	0.857
Cd40	Nfkbia	0.905

Cd40	Tnf	0.965
Cd40	Traf3	0.999
Csf1	Irf7	0.416
Csf1	Pik3r1	0.485
Csf1	Tlr9	0.491
Csf1	Tlr3	0.498
Csf1	Spp1	0.514
Csf1	Jun	0.53
Csf1	Nfkbia	0.543
Csf1	Il12b	0.552
Csf1	Fos	0.552
Csf1	Stat1	0.561
Csf1	Ifnb1	0.584
Csf1	Tlr2	0.623
Csf1	Mmp9	0.631
Csf1	Cxcl9	0.665
Csf1	Cxcl10	0.725
Csf1	Myd88	0.753
Csf1	Il1b	0.756
Csf1	Tnf	0.831
Cxcl10	Mmp9	0.635
Cxcl10	Map2k6	0.715
Cxcl10	Nfkbia	0.637
Cxcl10	Traf3	0.4
Cxcl10	Tnf	0.912
Cxcl10	Irf7	0.895
Cxcl10	Il1b	0.91
Cxcl10	Il12a	0.476
Cxcl10	Tlr2	0.817
Cxcl10	Tlr3	0.76
Cxcl10	Myd88	0.735
Cxcl10	Ticam2	0.45
Cxcl10	Spp1	0.474
Cxcl10	Ifna2	0.479
Cxcl10	Tlr5	0.537
Cxcl10	Jun	0.557
Cxcl10	Ikbke	0.58
Cxcl10	Tlr8	0.605
Cxcl10	Tlr9	0.699
Cxcl10	Ifi47	0.717
Cxcl10	Il12b	0.756
Cxcl10	Ifnb1	0.857
Cxcl10	Stat1	0.918
Cxcl10	Cxcl9	0.988
Cxcl9	Mmp9	0.52
Cxcl9	Tnf	0.826
Cxcl9	Irf7	0.708
Cxcl9	Il1b	0.796
Cxcl9	Tlr2	0.615
Cxcl9	Tlr3	0.585

Cxcl9	Myd88	0.563
Cxcl9	Ifnb1	0.667
Cxcl9	Stat1	0.883
Cxcl9	Tlr9	0.538
Cxcl9	Ifi47	0.719
Cxcl9	Tlr8	0.432
Cxcl9	Il12b	0.636
Fos	Mmp9	0.641
Fos	Nfkbia	0.685
Fos	Myd88	0.412
Fos	Tlr2	0.451
Fos	Spp1	0.468
Fos	Traf3	0.478
Fos	Irf7	0.528
Fos	Pik3r1	0.583
Fos	Il1b	0.678
Fos	Ifnb1	0.794
Fos	Stat1	0.864
Fos	Tnf	0.891
Fos	Mapk12	0.929
Fos	Mapk11	0.95
Fos	Jun	0.999
Ifi47	Irf7	0.8
Ifi47	Stat1	0.897
Ifna2	Tnf	0.41
Ifna2	Irf7	0.784
Ifna2	Il1b	0.409
Ifna2	Ifnb1	0.614
Ifna2	Stat1	0.764
Ifna4	Irf7	0.832
Ifna4	Ifnb1	0.645
Ifna4	Stat1	0.764
Ifna4	Ifna5	0.406
Ifna5	Irf7	0.728
Ifna5	Ifnb1	0.599
Ifna5	Stat1	0.685
Ifnb1	Mmp9	0.467
Ifnb1	Nfkbia	0.688
Ifnb1	Traf3	0.786
Ifnb1	Tnf	0.856
Ifnb1	Irf7	0.976
Ifnb1	Il1b	0.829
Ifnb1	Il12a	0.552
Ifnb1	Tlr2	0.814
Ifnb1	Tlr3	0.937
Ifnb1	Myd88	0.849
Ifnb1	Ikbke	0.84
Ifnb1	Tlr5	0.691
Ifnb1	Ticam2	0.719
Ifnb1	Tlr8	0.72

Ifnb1	Il12b	0.723
Ifnb1	Tlr9	0.841
Ifnb1	Jun	0.876
Ifnb1	Stat1	0.975
Ikbke	Nfkbia	0.779
Ikbke	Traf3	0.999
Ikbke	Tnf	0.975
Ikbke	Irf7	0.986
Ikbke	Il1b	0.619
Ikbke	Tlr2	0.732
Ikbke	Tlr3	0.882
Ikbke	Myd88	0.908
Ikbke	Tlr5	0.576
Ikbke	Jun	0.607
Ikbke	Tlr8	0.694
Ikbke	Tlr9	0.728
Ikbke	Ticam2	0.859
Ikbke	Stat1	0.94
Il12a	Tnf	0.692
Il12a	Il1b	0.881
Il12a	Tlr3	0.463
Il12a	Tlr9	0.513
Il12a	Myd88	0.551
Il12a	Tlr2	0.552
Il12a	Stat1	0.846
Il12a	Il12b	0.998
Il12b	Nfkbia	0.534
Il12b	Tnf	0.857
Il12b	Irf7	0.497
Il12b	Il1b	0.85
Il12b	Tlr2	0.732
Il12b	Tlr3	0.67
Il12b	Myd88	0.719
Il12b	Stat1	0.616
Il12b	Tlr9	0.712
Il12b	Jun	0.463
Il12b	Tlr5	0.521
Il12b	Tlr8	0.526
Il1b	Mmp9	0.874
Il1b	Nfkbia	0.902
Il1b	Traf3	0.781
Il1b	Map3k8	0.551
Il1b	Tnf	0.973
Il1b	Irf7	0.68
Il1b	Mapk12	0.53
Il1b	Spp1	0.628
Il1b	Tlr8	0.703
Il1b	Tlr5	0.724
Il1b	Pik3r1	0.73
Il1b	Mapk11	0.75

Il1b	Stat1	0.77
Il1b	Tlr9	0.793
Il1b	Tlr3	0.836
Il1b	Ticam2	0.842
Il1b	Tlr2	0.925
Il1b	Jun	0.962
Il1b	Myd88	0.987
Irf7	Nfkbia	0.57
Irf7	Traf3	0.962
Irf7	Tnf	0.721
Irf7	Tlr5	0.551
Irf7	Tlr8	0.721
Irf7	Tlr2	0.737
Irf7	Tlr9	0.838
Irf7	Ticam2	0.841
Irf7	Spp1	0.884
Irf7	Tlr3	0.895
Irf7	Jun	0.902
Irf7	Stat1	0.962
Irf7	Myd88	0.999
Jun	Mmp9	0.782
Jun	Map2k6	0.592
Jun	Nfkbia	0.937
Jun	Traf3	0.545
Jun	Map3k8	0.469
Jun	Tnf	0.989
Jun	Tlr2	0.668
Jun	Tlr3	0.679
Jun	Myd88	0.77
Jun	Ticam2	0.463
Jun	Stat1	0.838
Jun	Tlr9	0.55
Jun	Mapk11	0.975
Jun	Mapk12	0.976
Jun	Tlr5	0.463
Jun	Spp1	0.55
Map2k6	Traf3	0.492
Map2k6	Tnf	0.796
Map2k6	Mapk12	0.986
Map2k6	Mapk11	0.991
Map3k8	Nfkbia	0.588
Map3k8	Tlr3	0.42
Map3k8	Myd88	0.438
Map3k8	Tlr2	0.514
Map3k8	Tnf	0.593
Mapk11	Nfkbia	0.673
Mapk11	Tnf	0.792
Mapk11	Stat1	0.723
Mapk11	Mapk12	0.909
Mapk12	Nfkbia	0.541

Mapk12	Tnf	0.584
Mapk12	Stat1	0.76
Mmp9	Tlr3	0.403
Mmp9	Tlr9	0.421
Mmp9	Stat1	0.548
Mmp9	Myd88	0.552
Mmp9	Nfkbia	0.716
Mmp9	Spp1	0.73
Mmp9	Tlr2	0.742
Mmp9	Tnf	0.876
Myd88	Nfkbia	0.965
Myd88	Traf3	0.998
Myd88	Tnf	0.89
Myd88	Tlr2	0.99
Myd88	Tlr3	0.994
Myd88	Spp1	0.649
Myd88	Stat1	0.746
Myd88	Ticam2	0.843
Myd88	Tlr8	0.991
Myd88	Tlr5	0.997
Myd88	Tlr9	0.999
Nfkbia	Ticam2	0.43
Nfkbia	Tlr8	0.46
Nfkbia	Tlr5	0.515
Nfkbia	Tlr9	0.595
Nfkbia	Tlr3	0.636
Nfkbia	Stat1	0.726
Nfkbia	Traf3	0.737
Nfkbia	Tlr2	0.747
Nfkbia	Pik3r1	0.835
Nfkbia	Tnf	0.971
Pik3r1	Tlr2	0.835
Pik3r1	Stat1	0.531
Pik3r1	Spp1	0.57
Pik3r1	Tlr9	0.684
Spp1	Tnf	0.705
Spp1	Tlr2	0.595
Stat1	Traf3	0.63
Stat1	Tnf	0.942
Stat1	Tlr2	0.726
Stat1	Tlr3	0.826
Stat1	Tlr5	0.442
Stat1	Tlr8	0.539
Stat1	Tlr9	0.66
Ticam2	Traf3	0.969
Ticam2	Tnf	0.552
Ticam2	Tlr2	0.942
Ticam2	Tlr3	0.91
Ticam2	Tlr8	0.625
Ticam2	Tlr5	0.745

Ticam2	Tlr9	0.934
Tlr2	Traf3	0.641
Tlr2	Tnf	0.94
Tlr2	Tlr3	0.469
Tlr2	Tlr8	0.506
Tlr2	Tlr5	0.881
Tlr2	Tlr9	0.884
Tlr3	Traf3	0.914
Tlr3	Tnf	0.876
Tlr3	Tlr8	0.419
Tlr3	Tlr9	0.427
Tlr5	Traf3	0.544
Tlr5	Tnf	0.757
Tlr5	Tlr9	0.406
Tlr5	Tlr8	0.422
Tlr8	Traf3	0.636
Tlr8	Tnf	0.756
Tlr8	Tlr9	0.717
Tlr9	Traf3	0.759
Tlr9	Tnf	0.872
Tnf	Traf3	0.932
