# Supplementary material for: Transcriptomic Analysis of mRNA Expression Profiles in the Microglia of Mouse Brains Infected with Rabies Viruses of Varying Virulence
Source: Viruses. 2023 May 23;15(6):1223. doi: 10.3390/v15061223 (PMC10303246; doi:10.3390/v15061223)
Supplement: Supplementary file 1 [file viruses-15-01223-s001.zip › Supplymental materials20230522.pdf]

Supplementary Table S1. Quality Control of mRNA sequence

Sample	Bases_G	Q30_G	Raw_reads_M	Clean_reads_M	Map_ration_%	Q20_%	Q30_%
DMEM_4d	7.80	7.40	52.50	49.09	92.78	97.94	94.7
DMEM_4d	8.20	7.80	55.13	51.56	92.45	97.93	94.65
DMEM_4d	8.20	7.70	54.70	51.32	88.28	97.93	94.65
DMEM_7 d	8.20	7.70	54.85	51.21	88.34	97.9	94.67
DMEM_7 d	7.90	7.50	53.02	49.49	93.74	97.89	94.67
DMEM_7 d	7.90	7.40	52.69	49.23	92.49	97.99	94.84
rRC-HL_4d	7.60	7.20	50.78	47.47	93.22	98.11	94.95
rRC-HL_4d	8.20	7.80	54.84	51.27	92.68	98.04	94.85
rRC-HL_4d	7.90	7.55	53.01	46.35	93.79	98.1	94.99
rRC-HL_7d	6.80	6.52	45.43	42.86	94.82	98.11	94.99
rRC-HL_7d	8.10	7.62	54.17	50.38	94.94	97.62	93.81
rRC-HL_7d	6.90	6.63	46.65	43.61	93.62	98.04	94.85
GX074_4d	7.30	6.90	48.93	45.99	84.3	98.02	94.92
GX074_4d	7.90	7.40	53.06	49.67	89.59	967.63	93.71
GX074_4d	7.10	6.70	47.27	44.23	92.01	98.03	94.82
GX074_7 d	6.80	6.40	45.54	42.51	94.05	98.11	95.05
GX074_7 d	8.40	7.90	56.08	52.55	94.55	98.09	94.92
GX074_7d	8.40	7.90	56.14	52.24	94.89	98.09	94.94
CVS-24_4d	6.50	6.20	43.48	40.47	91.19	98.11	95.09
CVS-24_4d	8.10	7.71	54.17	50.68	94.96	98.05	94.93
CVS-24_4d	7.20	6.90	48.45	45.22	93.91	98.04	94.96
CVS-24_7d	6.50	6.20	43.73	40.86	91.97	98.13	95.14
CVS-24_7d	6.40	6.10	43.07	40.11	95.39	98.14	95.04
CVS-24_7d	7.80	7.40	52.32	49.21	94.41	98.97	94.68

Note:

Q30: The total number of bases with an accuracy of more than 99.9%;

Q20 %: Percentage of bases with more than 99% accuracy;

Q30 %: Percentage of bases with more than 99.9% accuracy.

Supplementary Table S4. Up -down DEGS

Control	Treat	UP-regulated	Down-regulated	Total
**DMEM_4**	**rRC_HL_4**	**1953**	**1669**	**3622**
**DMEM_4**	**GX074_4**	**158**	**107**	**265**
**DMEM_4**	**CVS24_4**	**2302**	**1795**	**4097**
**DMEM_7**	**rRC_HL_7**	**2557**	**2033**	**4590**
**DMEM_7**	**GX074_7**	**2667**	**2234**	**4901**
**DMEM_7**	**CVS24_7**	**3091**	**3246**	**6337**

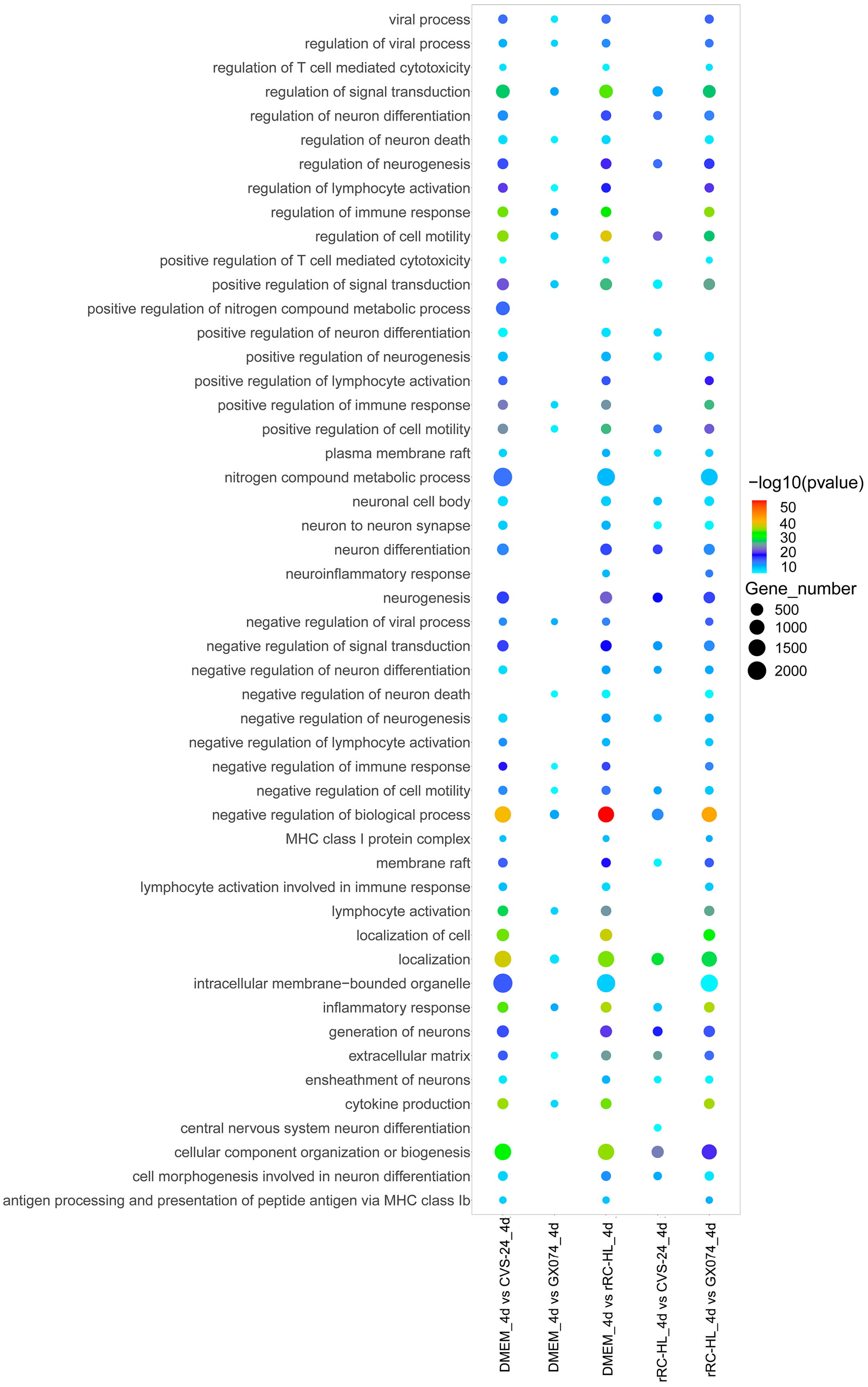

Supplementary Figure S1. GO term of mice brain infected RABV at 4 dpi

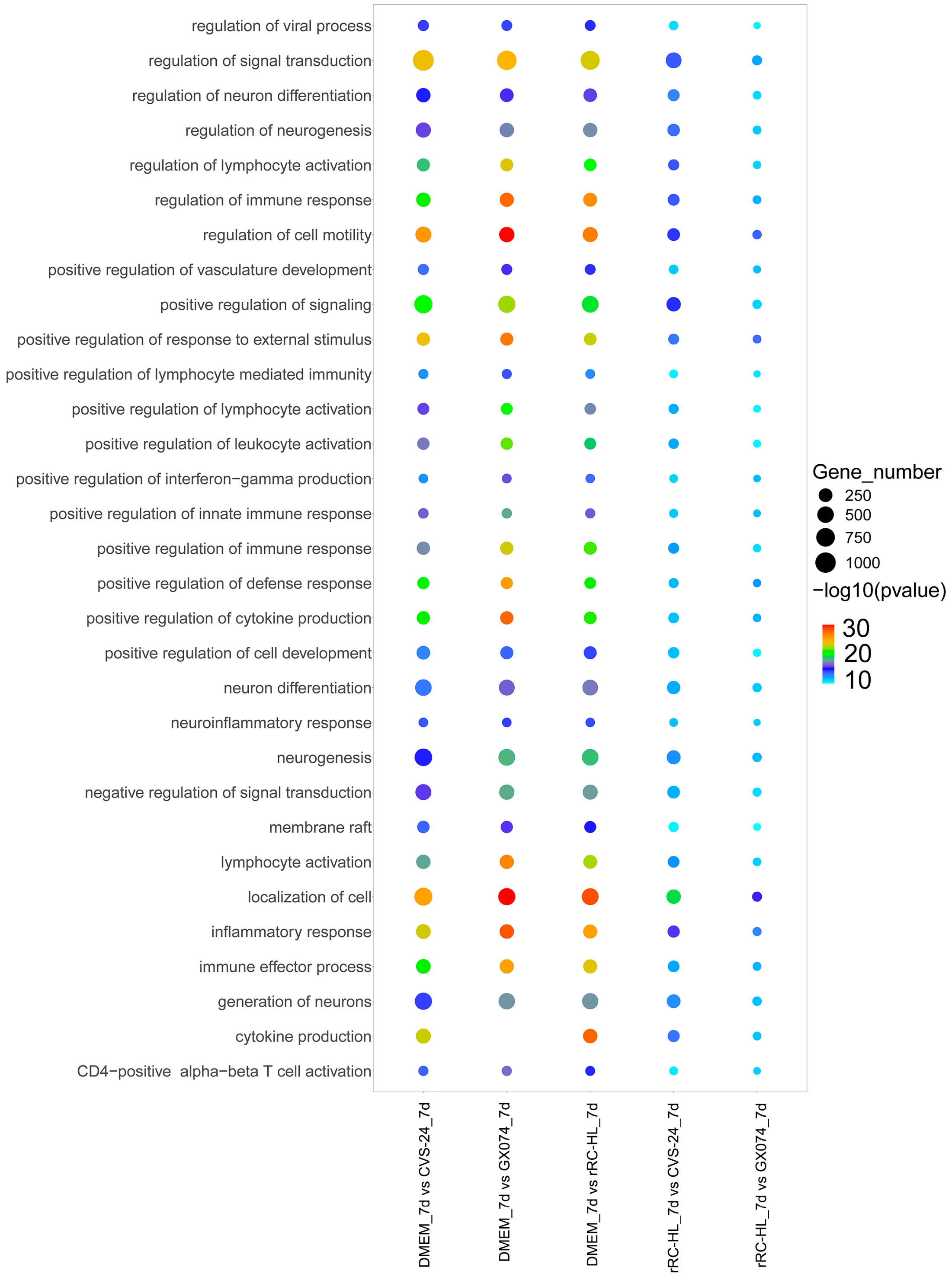

Supplementary Figure S2. GO term of mice brain infected RABV at 7 dpi

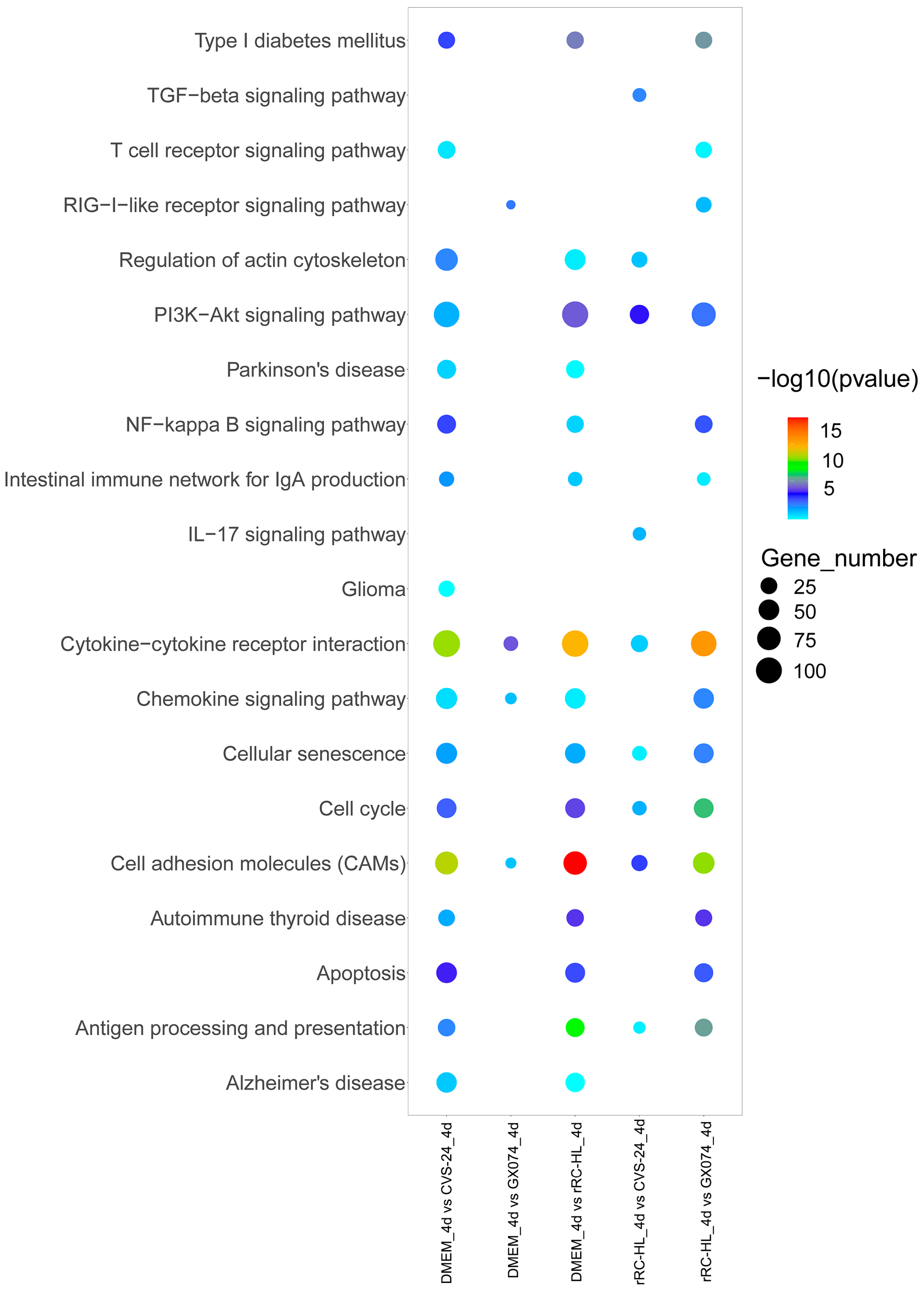

Supplementary Figure S3. KEGG analysis of microglia of mice brain infected with RABV at 4 dpi

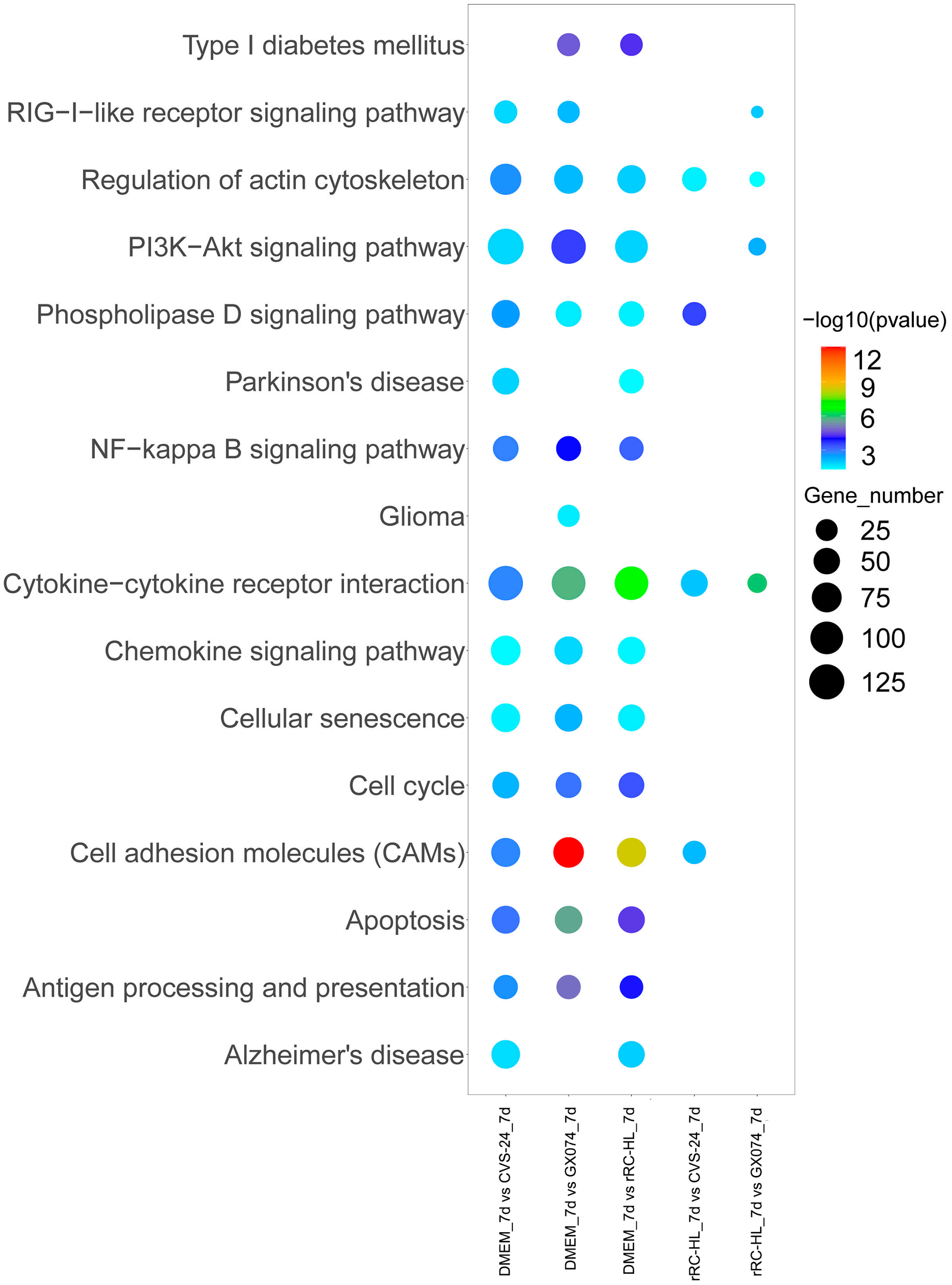

Supplementary Figure S4. KEGG analysis of microglia of mice brain infected with RABV at 7 dpi

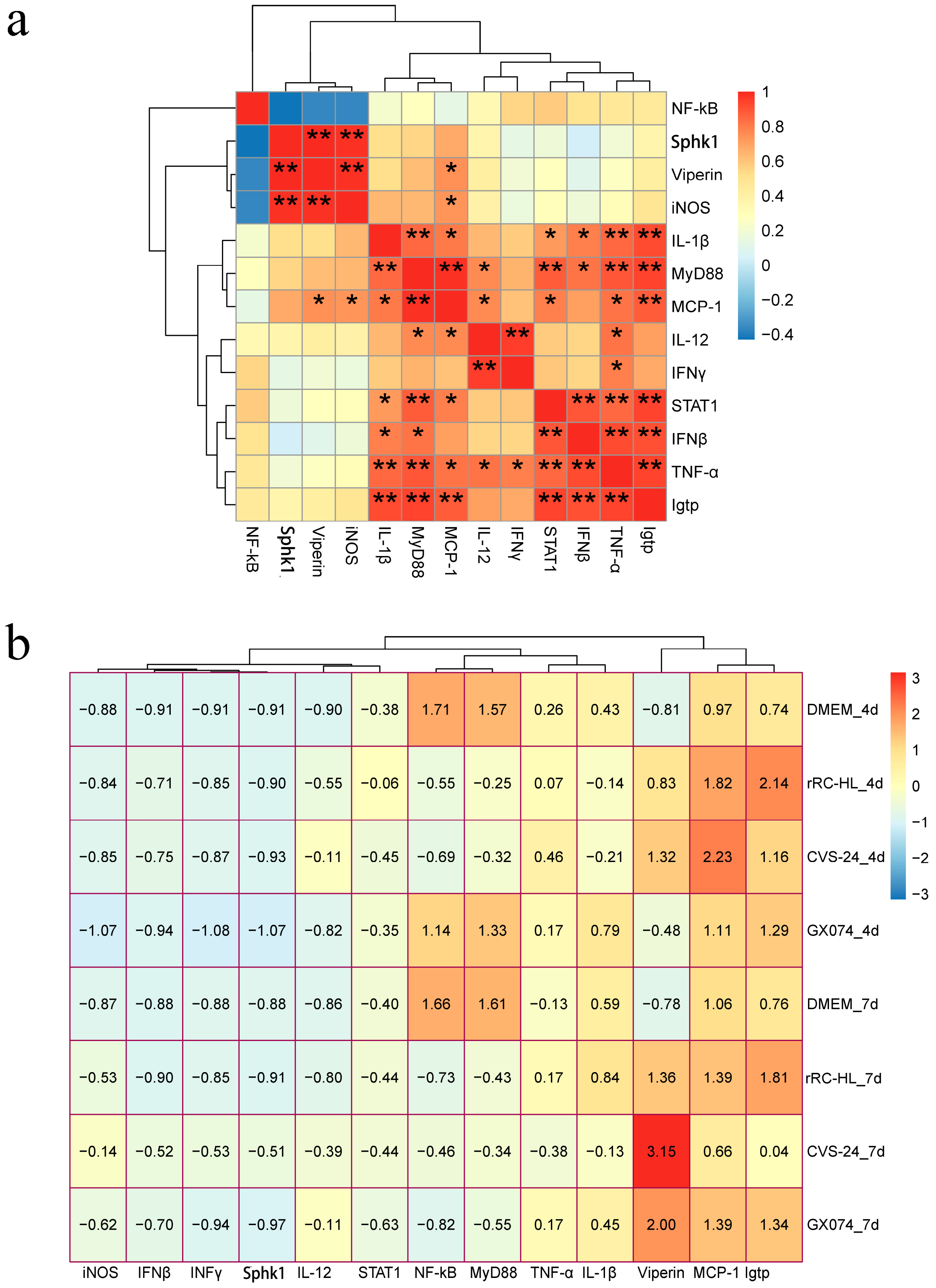
a
NF-kB
Sphk1
Viperin
iNOS
IL-1β
MyD88
MCP-1
IL-12
IFNγ
STAT1
IFNβ
TNF-α
Igtp
1
0.8
0.6
0.4
0.2
0
−0.2
−0.4
b
iNOS IFNβ INFγ Sphk1 IL-12 STAT1 NF-kB MyD88 TNF-α IL-1β Viperin MCP-1 Igtp
DMEM_4d −0.88 −0.91 −0.91 −0.91 −0.90 −0.38 1.71 1.57 0.26 0.43 −0.81 0.97 0.74
rRC-HL_4d −0.84 −0.71 −0.85 −0.90 −0.55 −0.06 −0.55 −0.25 0.07 −0.14 0.83 1.82 2.14
CVS-24_4d −0.85 −0.75 −0.87 −0.93 −0.11 −0.45 −0.69 −0.32 0.46 −0.21 1.32 2.23 1.16
GX074_4d −1.07 −0.94 −1.08 −1.07 −0.82 −0.35 1.14 1.33 0.17 0.79 −0.48 1.11 1.29
DMEM_7d −0.87 −0.88 −0.88 −0.88 −0.86 −0.40 1.66 1.61 −0.13 0.59 −0.78 1.06 0.76
rRC-HL_7d −0.53 −0.90 −0.85 −0.91 −0.80 −0.44 −0.73 −0.43 0.17 0.84 1.36 1.39 1.81
CVS-24_7d −0.14 −0.52 −0.53 −0.51 −0.39 −0.44 −0.46 −0.34 −0.38 −0.13 3.15 0.66 0.04
GX074_7d −0.62 −0.70 −0.94 −0.97 −0.11 −0.63 −0.82 −0.55 0.17 0.45 2.00 1.39 1.34
3
2
1
0
−1
−2
−3

Supplementary Figure S5. The functional analysis of gene expression

(a) Pearson coefficients, red color represents positive correlation, blue color represents negative correlation, *indicates significant positive correlation and significant negative correlation. *p<0.05; **p<0.01. (b) Difference between multiple analysis. Red color was high expression, and blue was low expression.
